# Comparison of Treponema-specific immunoglobulin G (IgG) index with Treponema pallidum particle agglutination (TPPA) index for detection of intrathecal Treponema-specific antibody synthesis for serological diagnosis of neurosyphilis

**DOI:** 10.3205/id000081

**Published:** 2023-08-17

**Authors:** Nele Wellinghausen, Andrea Götz, Teresa Esthela Rangel Vivar

**Affiliations:** 1 MVZ Labor Ravensburg, Germany

**Keywords:** neurosyphilis, intrathecal antibody synthesis, TPPA index, Treponema

## Abstract

The determination of *Treponema*-specific intrathecal immunoglobulin synthesis with the *Treponema* *pallidum* particle agglutination (TPPA) index is a well-established method recommended in German guidelines for the diagnosis of neurosyphilis. However, the TPPA test is no longer available. The aim of this study was to evaluate whether the determination of a *Treponema*-specific immunoglobulin G (IgG) index can substitute the TPPA index. Serum and cerebrospinal fluid (CSF) samples from patients with confirmed (n=6) and probable (n=3) neurosyphilis as well as patients with adequately treated syphilis without neurosyphilis (n=4) were investigated. In addition to index calculation further CSF parameters were determined. The results of the *Treponema* IgG and the TPPA index were consistent in all patients with confirmed neurosyphilis and non-neurosyphilis patients. In two patients with probable neurosyphilis the IgG index appeared more plausible than the TPPA index when taking into account all available laboratory and clinical data of the patients. In conclusion, the determination of *Treponema*-specific intrathecal immunoglobulin synthesis with the IgG index appears to be a suitable alternative to the TPPA index.

## Introduction

*Treponema* *pallidum* is the causative agent of syphilis, a sexually transmitted infection with increasing incidence in Europe [1]. Syphilis can involve several organ systems as well as the central nervous system. Meningitis, meningovascular syphilis, cranial nerve palsies, and ophthalmic or otologic involvement are clinical manifestations of early neurosyphilis while late neurosyphilis mainly encompasses tabes dorsalis, paralytic neurosyphilis, and the appearance of syphilitic gummas. Confirmation or exclusion of neurosyphilis is essential to ensure adequate antibacterial therapy for the patients.

The diagnosis of neurosyphilis relies on both clinical and laboratory diagnostic criteria including the investigation of cerebrospinal fluid (CSF) [2], [3]. Apart from the serological confirmation of syphilis by a positive *Treponema*-specific antibody test (fluorescent *Treponema pallidum* absorption test (FTA-abs test), immunoassay, *Treponema pallidum* particle or hem agglutination assay (TPPA/TPHA) etc.) in serum, the detection of *Treponema*-specific antibodies in CSF are mandatory for the laboratory confirmation of neurosyphilis [2], [3]. According to the German diagnostic guidelines, the diagnosis of neurosyphilis is made from a combination of clinical findings, CSF parameters, and the detection of intrathecal synthesis of antibodies against *Treponema* [3]. For the diagnosis of proven neurosyphilis the latter can be confirmed by a positive CSF/serum TPPA/TPHA index or FTA-abs-test index [4]. Syphilis patients who fulfill clinical and laboratory criteria but miss a positive *Treponema*-specific CSF/serum antibody index are regarded as probable neurosyphilis [3], [4]. Unfortunately, the TPPA is no longer available on the market since the manufacturer stopped its production in 2022, and availability of the TPHA test has also temporarily been limited. 

Very recently, the determination of intrathecal synthesis of *Treponema*-specific immunoglobulin G (IgG) by recombinant enzyme-linked immunosorbent assay (ELISA) has been introduced as a new and promising tool for diagnosis of neurosyphilis [5]. Therefore, we compared the TPPA index with the *Treponema* IgG index in patients with confirmed neurosyphilis (n=6), probable neurosyphilis (n=3), and adequately treated past syphilis without neurosyphilis (n=4) according to German guidelines in order to evaluate whether the *Treponema* IgG index can substitute the TPPA index as a diagnostic tool for the diagnosis of neurosyphilis. 

## Methods

All serum samples were sent to our laboratory for diagnostic work-up of syphilis infection with informed consent to all performed diagnostic tests. TPPA was determined in parallel in serum and CSF with the test of Fujirebio (Tokio, Japan), and CSF/serum index was calculated according to Reiber’s method [6]. A TPPA index ≥3.0 indicates intrathecal *Treponema*-specific antibody synthesis. *Treponema-pallidum*-specific IgG was determined in serum and CSF by *Treponema* IgG ELISA (EI 2111-9601-L G, Euroimmun, Luebeck, Germany, using recombinant *Treponema pallidum* proteins) and in parallel on the EUROLab workstation ELISA (Euroimmun). Serum and CSF samples were analyzed in standard dilutions of 1:404 and 1:2, respectively, on the same microtiter plate according to the recommendations of the manufacturer and, if necessary, pre-diluted up to 1:40 (serum) and 1:200 (CSF) to obtain optical density values within the linear range of the standard curve. CSF/serum index was calculated according to Reiber’s method [6]. Albumin and IgG in serum and CSF was determined by nephelometry on a BN-2 system (Siemens Healthcare, Eschborn, Germany). A *Treponema* IgG index ≥1.5 indicates intrathecal *Treponema*-specific IgG synthesis. The titer of rapid plasma reagin was determined with the test by Becton Dickinson (Sparks, MD, USA). A titer ≥4 in serum and ≥1 in CSF is considered positive. Total protein was determined in CSF on the Advia 1800 system (Siemens Healthineers, Erlangen, Germany), reference range 0.15–0.45 g/l. chemokine (C-X-C motif) ligand 13 (CXCL13) was determined by ELISA (Euroimmun, Luebeck, Germany) on an EUROAnalyzer. A concentration >20 pg/ml is defined as positive according to the manufacturer. CSF was rethawed twice after freezing at –20°C; thus, concentrations of CXCL13 may be false low in some cases. *Borrelia*-specific IgG in serum and CSF was determined by chemiluminescence immuno assay on the Liaison XL system (Diasorin, Dietzenbach, Germany), and dilutions for the determination of the index were performed by the analyzer. 

## Results

In all of the six patients with confirmed neurosyphilis both the TPPA index and the *Treponema* IgG index were pathologically increased (Table 1 (Tab. 1), Table 2 (Tab. 2) and Table 3 (Tab. 3)). All patients also had a markedly elevated TPPA titer in CSF and, interestingly, all patients (except one with a missing amount of CSF for measurement) also showed an increased concentration of CXCL13 in CSF (Table 1 (Tab. 1)).

The three patients with probable neurosyphilis had normal TPPA indices, but two of them showed an increased *Treponema* IgG Index >1.5 (patient no. 7 and 9) and one had a borderline index of 1.48 (patient no. 8, Table 2 (Tab. 2)). In patient no. 7 with a *Treponema* IgG index of 1.97, further serological and CSF results were highly suggestive of neuroysphilis, including a positive RPR in serum and CSF, a CSF TPPA titer of 1,024, CSF pleocytosis, and increased CXCL13 >5,000 pg/ml (Table 1 (Tab. 1) and Table 2 (Tab. 2)). Clinically a newly manifested monocular scotoma led to the diagnosis of active syphilis infection in patient no. 7. Patient no. 9 showed an increased *Treponema* IgG index of 1.89, but the TPPA index was normal. The patient presented for the first time with active syphilis infection, and increased CSF total protein as well as borderline intrathecal IgM synthesis favour diagnosis of neurosyphilis but TPPA titer below 640 in CSF and absence of pleocytosis are contradictory. Nevertheless, the involvement of the meninges or the central nervous system appears possible. Patient no. 8 presented with meningo-encephalitic symptoms, and diagnostic workup for syphilis was initiated due to a CXCL13 concentration >5000 pg/ml after neuroborreliosis, cryptococcosis, carcinomatous meningitis, and CNS lymphoma had been ruled out. 

Four patients with adequately treated past syphilis infection were included in the study. They received lumbar puncture for other reasons. One patient had acute tick-borne encephalitis (patient no. 10). All patients showed normal a TPPA index and *Treponema* IgG index, and TPPA, RPR and CXCL13 levels in CSF were also unremarkable (Table 1 (Tab. 1) and Table 2 (Tab. 2)). In addition, five patients with neuroborreliosis and positive intrathecal *Borrelia*-specific IgG synthesis (IgG index 2.0 to 4.5, median 4.1) were investigated. *Treponema* IgG were not detectable in serum or CSF in any of these patients. Unfortunately the TPPA index could not be determined since the reagent was not available anymore. 

## Discussion

The results of the *Treponoema* IgG index and the TPPA index were consistent in all patients with confirmed neurosypilis as well as syphilis patients without neurosyphilis. Discrepant results between *Treponema* IgG index and TPPA index occurred only in patients with a probable diagnosis of neurosyphilis. In these cases the IgG index was markedly lower (<2.0) in comparison to the patients with confirmed neurosyphilis (15.6–588). The cutoff value for positive intrathecal *Treponema* IgG synthesis is ≥1.5 according to the manufacturer, but Alberto et al. suggested a cutoff of 1.7 by receiver operating characteristic (ROC) analysis resulting in a sensitivity of 59% and a specificity of 98.6% for diagnosis of neurosyphilis [5]. Thus, these increased indices were still above the cutoff proposed by Alberto et al. Results of TPPA in CSF, RPR in CSF, and CXCL13 were also noticeable in the discrepant patients. A CSF TPPA titer ≥640 has a high specificity for neurosyphilis [7], [8], [9] and constitutes a diagnostic criterion for neurosyphilis in British guidelines [10]. According to European and other international guidelines, the detection of cardiolipin antibodies in CSF is also indicative of neurosyphilis [1], but this parameter has not been included in the German guidelines. CXCL13 has recently been published as a diagnostic marker for neurosyphilis. A cutoff of 184 pg/ml was determined by ROC analysis to have a sensitivity of 82.8% and a specificity of 81.1% for symptomatic syphilitic meningitis [7], while others found a sensitivity and specificity of 41% and 79%, respectively, for a cutoff of 250 pg/ml [11]. In our experience, apart from neurosyphilis CXCL13 >5000 pg/ml is only seen in neuroborreliosis, cryptococcosis, and carcinomatous meningitis or CNS lymphoma, and these differential diagnoses have been ruled out in all three patients. Taking into account all available data of CSF analysis in the discrepant patients as well as recommendations of international guidelines, the diagnosis of neurosyphilis appears likely in these patients. 

## Conclusion

In conclusion, our data suggest that the determination of the *Treponema* IgG index by ELISA appears to be an adequate substitute for the former TPPA index for the diagnosis of neurosyphilis. In addition, it is easy to perform both manually as well as on an automated ELISA plate processor. Nevertheless, a limitation of our study is that the number of samples from patients with neurosyphilis was small; however, the investigation of further patients is not possible since the TPPA test is no longer available on the market. 

## Notes

### Competing interests

The authors declare that they have no competing interests.

### Acknowledgments

We thank Martina Graf for excellent technical assistance and Dirk Suehnel and Bjoern Oswald for the helpful discussion of the results. We thank Professor Hans-Jochen Hagedorn for the critical review of the manuscript and for his helpful comments.

## Erratum

In the article the TPPA index was calculated according to the German guidelines [3] by using the values of TPPA and Q_IgG_. However, in patients no. 1 to 6 the Q_IgG_ was greater than the Q_lim_ and, thus, the Q_lim_ should be used. The corrected TPPA indices are shown in the corrected Tab. 1. In addition, in patient 2 the *Treponema* IgG index was calculated falsely and was corrected. Furthermore, patients no. 6 and 9 had a blood-CSF barrier dysfunction, and patient no. 12 hat a borderline blood-CSF barrier dysfunction.

In Tab. 2 the values of Q_IgG_ Q_Albumin_ and Q_lim_ for patient no. 8 were mistakenly shown as those of patient no. 7. The corrected values of patient no. 8 are shown in the corrected Tab. 2. 

We added a table depicting the measured concentrations of *Treponema* IgG in serum and CSF as well as the final dilution factors used for measurement of serum and CSF, respectively (Tab. 3). Diluted serum and CSF samples resulted in *Treponema* IgG concentrations within the standard curve of the ELISA test, i.e. between 5 U/ml and 100 U/ml. *Treponema* indices were calculated by the formula of Reiber et al. [6] within our laboratory information system considering the Q_lim_ if Q_IgG_>Q_lim_. 

The values in the third sentence of the discussion were amended.

Furthermore, Professor Hagedorn was added in the acknowledgements.

## Figures and Tables

**Table 1:**
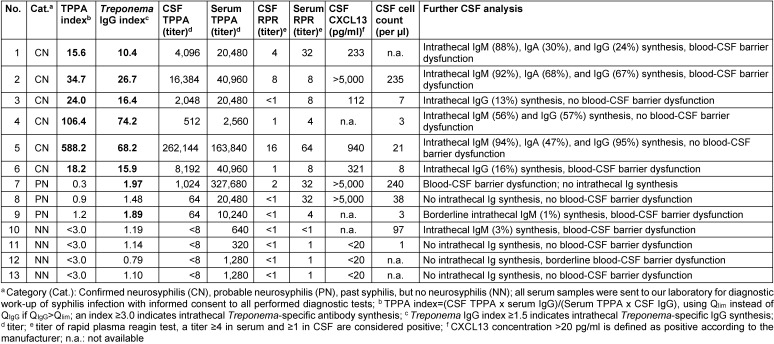
Clinical data of the patients and results of the *Treponema* diagnostics

**Table 2:**
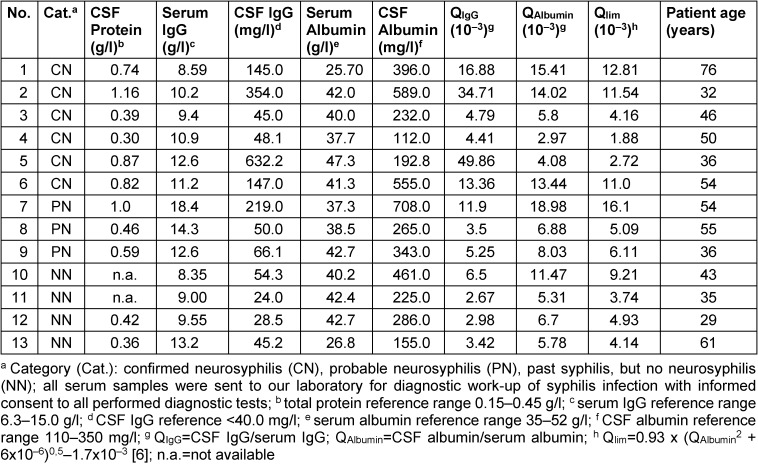
Results of CSF protein and Reiber diagram analysis in the patients

**Table 3:**
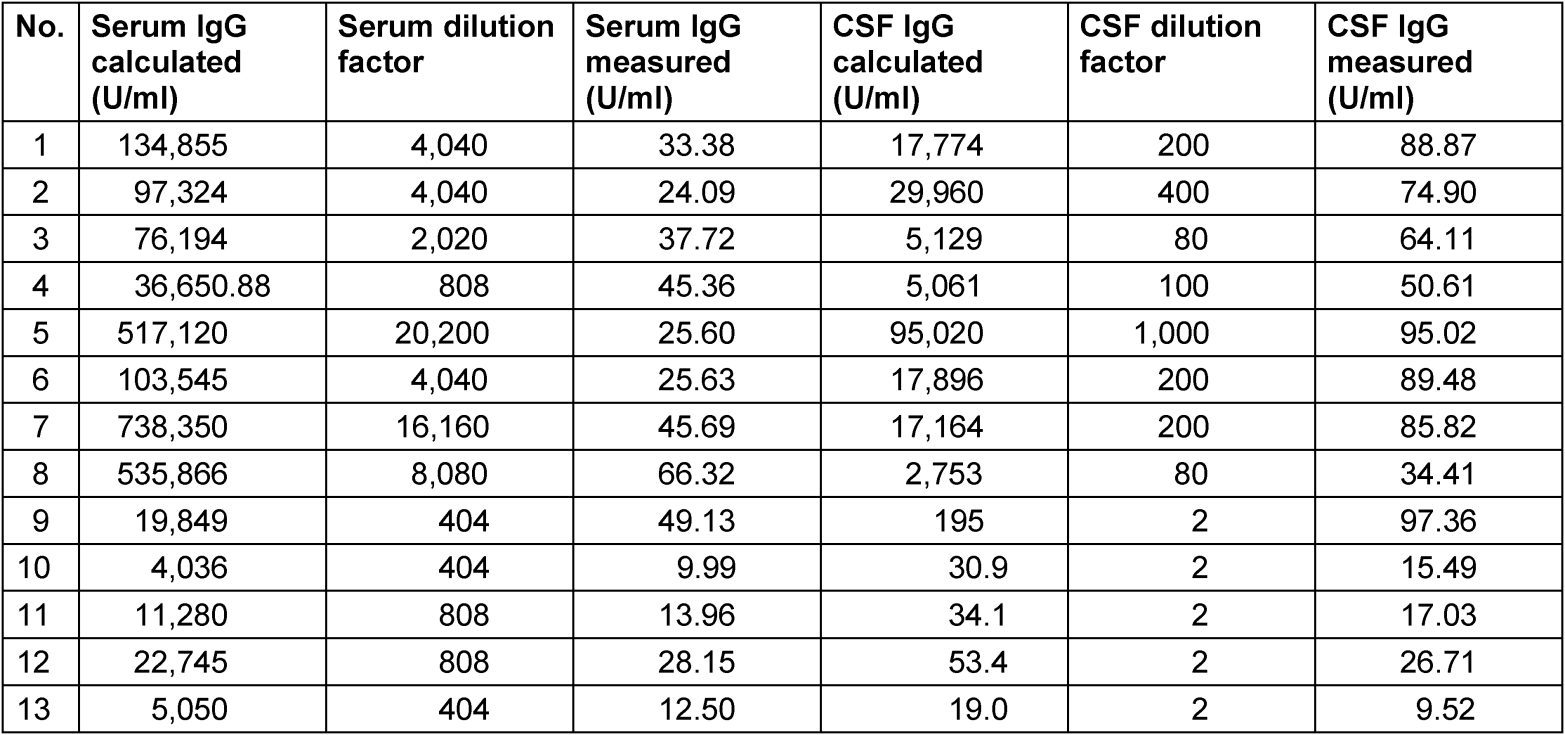
Raw data of *Treponema* IgG ELISA and dilution factors used in serum and CSF
